# Mucinous Adenocarcinoma Arising From Teratoma Years After Testicular Nonseminomatous Germ Cell Tumor

**DOI:** 10.7759/cureus.91983

**Published:** 2025-09-10

**Authors:** Hoon Oh, Neud Kiros, Sayf Al-Katib

**Affiliations:** 1 Diagnostic Radiology, Oakland University William Beaumont School of Medicine, Rochester Hills, USA; 2 Diagnostic Radiology, Corewell Health William Beaumont University Hospital, Royal Oak, USA

**Keywords:** growing teratoma syndrome, long-term surveillance, mature teratoma, mucinous adenocarcinoma, nonseminomatous germ cell tumor, retroperitoneal mass, somatic-type malignancy, surgical resection, testicular cancer, tumor transformation

## Abstract

Growing teratoma syndrome (GTS) is a rare complication of nonseminomatous germ cell tumors (NSGCTs). We present a case of a patient with a remote history of NSGCT, previously treated with orchiectomy and chemotherapy, who developed a large retroperitoneal mass over two decades later. The patient had normal tumor markers and multiple inconclusive biopsies. Surgical excision was performed, and the pathology returned as mucinous adenocarcinoma arising within a teratoma. This case highlights both the critical importance of long-term surveillance following NSGCT and the need for complete surgical resection of residual teratomatous disease even years after initial treatment due to the risk of delayed malignant transformation.

## Introduction

Growing teratoma syndrome (GTS) is a rare but well-recognized complication of nonseminomatous germ cell tumors (NSGCTs). It is defined by the paradoxical enlargement of metastatic or residual tumor masses despite normalization of serum tumor markers following treatment, with histopathology revealing only mature teratoma components [1,2]. Although histologically benign, the mature teratoma behaves in a clinically aggressive manner and is resistant to both chemotherapy and radiotherapy, often necessitating surgical excision [3,4].

In post-pubertal males, mature teratomas are considered malignant due to their derivation from transformed germ cell elements and their potential for recurrence, local invasion, and malignant transformation [2,3]. This is in contrast to prepubertal teratomas, which are typically benign. The malignant potential of post-pubertal mature teratomas is supported by cytologic atypia, genomic instability, and documented cases of transformation into somatic-type malignancies, including sarcoma, primitive neuroectodermal tumor, and adenocarcinoma [3-5].

The risk of this somatic-type transformation is unknown but has been estimated at 3-6% and is associated with increased morbidity and mortality. In such cases, surgical resection is both curative and essential. Delayed excision of a recurrent retroperitoneal mass theoretically increases the risk of secondary malignancy and local progression [4].

We present the case of a patient with a history of NSGCT who developed a large retroperitoneal mass nearly two decades after initial treatment. Pathology revealed mucinous adenocarcinoma arising within a teratoma. Our report underscores the importance of aggressive surgical management and long-term surveillance in patients with residual teratomatous disease, even when tumor markers remain within normal limits.

## Case presentation

**Figure 1:**
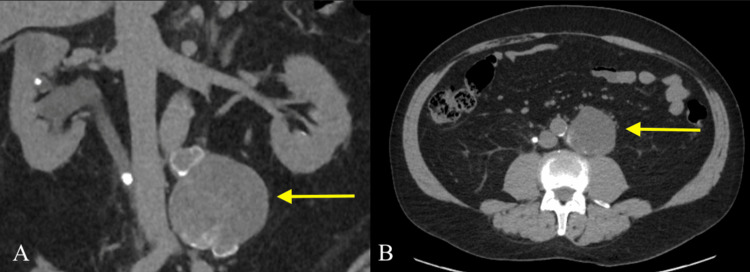
Coronal and axial non-contrast CT images of the abdomen and pelvis. Coronal image (A) demonstrates mild hydronephrosis of the right kidney secondary to an obstructing 9 mm calculus in the mid-right ureter. Incidentally, detected a 7.7 cm partially calcified mass in the left periaortic retroperitoneum is seen on axial image (B) as well (arrows).

A 62-year-old male with a history of kidney stones and a remote history of left testicular cancer, status post orchiectomy and chemotherapy in 2003, presented to the emergency room in January 2024 with right flank pain that had developed the night prior. The pain lasted roughly four hours and had largely subsided by the time of presentation. In the emergency room, he was afebrile and hemodynamically stable. Non-contrast computed tomography (CT) of the abdomen and pelvis demonstrated an obstructing 9 mm calculus in the mid-portion of the right ureter with resultant mild hydronephrosis. Incidentally, a 7.7 cm partially calcified mass in the left periaortic retroperitoneum was noted (Figure 1), which was new compared to a CT performed 10 years prior. Given his history of testicular cancer, the differential included metastatic disease or a primary cystic retroperitoneal tumor. Urologic evaluation and contrast-enhanced magnetic resonance imaging (MRI) were recommended.

Regarding his oncologic history, he was diagnosed with a left testicular nonseminomatous germ cell tumor at the time of radical left orchiectomy in 2003. A staging CT showed retroperitoneal lymphadenopathy, for which he was treated with cisplatin, etoposide, and bleomycin for three cycles and achieved complete remission.

The patient was discharged from the emergency department and scheduled for follow-up with urology and oncology early the next week. Tumor markers, including alpha-fetoprotein (AFP), beta human chorionic gonadotropin (β-hCG), and lactate dehydrogenase (LDH), were ordered and were within normal limits.

**Figure 2:**
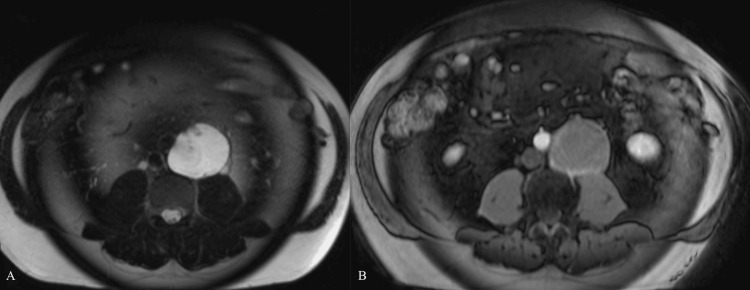
Axial MRI images of the abdomen T2 (A) and T1 post-contrast (B) MRI images of the abdomen demonstrate a well-circumscribed, T2-hyperintense retroperitoneal mass with thin peripheral rim enhancement without internal nodularity or solid enhancement. MRI: Magnetic resonance imaging

MRI demonstrated a markedly T2-hyperintense cystic mass with thin peripheral rim enhancement and no significant internal enhancement or nodularity (Figure 2). Pathology from a CT-guided biopsy showed mucin. A repeat biopsy was performed, and minute mucin-like material was obtained. After consultation with the patient’s urologist and oncologist, the decision was made to pursue operative management via excision of the mass and spermatic cord with retroperitoneal lymph node dissection. The patient’s urologist suspected a growing teratoma.

Pathology from the dominant mass revealed pools of dissecting mucin with focally dysplastic glandular epithelium with goblet cell morphology, compatible with mucinous adenocarcinoma. This was suspected to be arising from a teratoma within the dominant mass due to metastatic adenocarcinoma and cytologically benign epithelium with squamous differentiation identified within a retroperitoneal lymph node.

## Discussion

Teratomas are tumors that arise from germ cells and contain more than one embryonic germ cell layer [6]. The pathogenesis of growing teratoma syndrome (GTS) is not fully understood. The prevailing hypotheses regarding the etiology of GTS include proliferation of mature, differentiated cells inherently resistant to chemotherapy, or chemotherapy-induced alterations in cellular kinetics that promote transformation into mature teratoma [1,7]. Complete surgical excision is the mainstay of treatment, as mature teratomas are resistant to chemotherapy and radiotherapy, and incomplete resection increases the risk of recurrence and malignant transformation. Prognosis is generally excellent following curative resection, with long-term survival exceeding 90% in most series [8].

Testicular cancer is highly curable, with five-year survival rates exceeding 90% across all stages due to surgery and cisplatin-based chemotherapy [9]. Follow-up protocols are stage-specific and account for the risk of growing teratoma syndrome, emphasizing early detection and surgical excision of residual masses, as these are resistant to further chemotherapy [10].

Growing teratoma syndrome is an uncommon pathology that illustrates the balance an effective radiologist must maintain. When interpreting findings that do not have a clear etiology, offering a broad differential diagnosis is a useful way to avoid conveying a false sense of assurance. Conversely, when a diagnosis can be suggested with reasonable confidence, it should be identified as the leading consideration to help prevent unnecessary additional testing. In a patient with a prior cancer history, recurrent or metastatic disease warrants serious consideration. Without the history of prior testicular cancer, non-neoplastic possibilities in this case could include rare or atypical presentations of common entities such as sequelae of prior retroperitoneal hemorrhage, pancreatic pseudocyst, lymphatic malformation, multicystic peritoneal mesothelioma, or hydatid cyst.

Additionally, suggesting a biopsy should ideally be reserved for findings that truly warrant further investigation and are expected to impact clinical decision-making. In the context of testicular cancer surveillance, the American Urological Association advises that invasive procedures be pursued only when imaging or clinical findings are suspicious and the results would alter management. Late recurrences, while uncommon, have been documented in long-term follow-up [11]. A biopsy was an appropriate next step in management, and a follow-up biopsy after mucin was obtained was also justified. Assessing the concordance of biopsy results with imaging findings and performing radiologic-pathologic correlation is standard in mammography and, although not required in most practices, is an essential component of both understanding the imaging appearances of various disease processes and auditing the rate of negative biopsies.

Although numerous conditions can cause enlarging or radiologically new masses in patients with a history of nonseminomatous germ cell tumor (NSGCT), recognizing growing teratoma syndrome is essential to avoid unnecessary chemotherapy when recurrence or treatment failure is incorrectly presumed.

A high index of suspicion can also avoid complicated surgical excision if recognized promptly. Our case of dedifferentiation into mucinous adenocarcinoma highlights the potential risk faced by patients lost to follow-up. Had the patient not developed obstructive uropathy, it is unclear when he would have presented it and whether his tumor would have remained resectable, given its proximity to the abdominal aorta.

## Conclusions

Growing teratoma syndrome is a rare complication of nonseminomatous germ cell tumors. Awareness of this condition is important for two reasons: to prevent unnecessary chemotherapy and to expedite appropriate treatment with surgical excision. In patients with a history of nonseminomatous germ cell tumors, long-term radiologic and serologic surveillance are essential components of comprehensive care. Prompt recognition is particularly critical given the potential for delayed malignant transformation, even decades after initial treatment.
